# Development and Performance Evaluation of a Novel Epoxy-Modified Bitumen for Large-Void Porous Asphalt Concrete (LV-PAC)

**DOI:** 10.3390/polym18080916

**Published:** 2026-04-09

**Authors:** Xing Huang, Dongwei Cao, Qian Zhou, Changjing Xu, Hongmei Wei, Wentao Yang, Mingming Zhang

**Affiliations:** 1 School of Traffic and Transportation, Chongqing Jiaotong University, Chongqing 400074, China; 611240110021@mails.cqjtu.edu.cn; 2 Quanzhou Pingbu Engineering Technology Co., Ltd., Quanzhou 362000, China; 3 Research Institute of Highway Ministry of Transport, Beijing 100088, China; 4 School of Civil Engineering, Chongqing Jiaotong University, Chongqing 400074, China; 622240951035@mails.cqjtu.edu.cn; 5 School of Materials Science and Engineering, Chang’an University, Xi’an 710064, China; zmm@chd.edu.cn; 6 Shandong Hi-Speed Infrastructure Construction Co., Ltd., Jinan 250098, China; 7 Key Laboratory of Highway Engineering Technology in Arid and Desert Areas, Operation Branch of Xinjiang Communications Investment (Group) Co., Ltd., Urumqi 830000, China; 8 Zhejiang Scientific Research Institute of Transport, Hangzhou 311300, China

**Keywords:** large-void porous asphalt concrete, epoxy bitumen, pavement performance, pavement functional characteristics, engineering application

## Abstract

To address the limited drainage capacity of conventional porous asphalt pavements under high-intensity rainfall, this study proposes the use of epoxy-modified bitumen to develop a large-void porous asphalt concrete (LV-PAC) with a target air void content of 25%. This approach represents a novel application of epoxy-modified bitumen to enhance permeability in porous pavement systems. The LV-PAC exhibited improved high-temperature stability, permeability, and clogging recovery capability compared with a conventional high-viscosity porous asphalt concrete (HV-PAC), though its low-temperature deformation capacity was relatively lower. All evaluated performance indicators met the required specifications, highlighting the potential of epoxy-modified bitumen for use in large-void porous pavements pending further field validation.

## 1. Introduction

Porous asphalt pavement (PAP) is an open-graded asphalt concrete surface course structure designed based on a skeleton-embedded gradation. Featuring an interconnected pore network, this pavement facilitates rapid rainwater infiltration [1,2]. Furthermore, it serves a dual drainage function combining vertical percolation and lateral diversion, driven by the hydraulic potential gradient formed through cross-slope design. This system effectively mitigates surface water runoff during rainfall events [3,4,5]. Porous asphalt pavement is widely implemented in expressways globally owing to its functional benefits, including effective drainage, noise reduction, and enhanced skid resistance. However, existing comprehensive technical frameworks for PAP predominantly utilize polymer-modified high-viscosity bitumen requiring construction temperatures exceeding 180 °C [6,7,8]. Consequently, current applications are largely confined to regions with mean annual rainfall below 1500 mm and lower rainfall intensity. The elevated construction temperature accelerates bitumen aging and inevitably entails substantial energy consumption and increased carbon emissions, contradicting China’s national ‘Dual Carbon’ strategy (carbon peak and carbon neutrality) [9]. Therefore, for regions experiencing mean annual rainfall exceeding 1500 mm and high rainfall intensity, there is a need to develop durable large-void porous asphalt pavement solutions to better exploit their drainage potential.

Epoxy-modified bitumen forms an irreversible, high-strength, three-dimensional cross-linked network upon curing [10,11,12]. This renders it highly suitable for pavement applications demanding superior bitumen performance characteristics [13]. Jiang et al. [14] fabricated a hot-mix epoxy bitumen and subsequently designed hot-mix porous epoxy asphalt concrete with target air void contents ranging from 16% to 24%. The experimental results demonstrated that for polymer-modified bitumen cured using a two-stage process (160 °C for 2 h followed by 60 °C for 4 days) and an increase in air void content led to reductions in the epoxy asphalt concrete’s high-temperature performance, freeze–thaw resistance, moisture–heat damage resistance, and dynamic modulus. The low-temperature performance of HPEC exhibited an inflection point at a target air void content of 20%. Wang et al. [15] investigated epoxy-modified bitumen incorporating polyethylene glycol (PEG) of varying molecular weights (M_n_) and applied it within recycled porous asphalt pavement (RAP). The results indicated that single-factor analysis of variance (ANOVA) revealed that the M_n_ of PEG significantly influenced various key physical and functional parameters of the RAP-containing asphalt concrete. Consequently, PEG-modified EA utilizing PEG with an M_n_ ranging between 800 and 1000 g/mol is recommended as the binder for porous structures incorporating RAP. Luo et al. [16] utilized epoxidized soybean oil (ESBO) to develop a bio-based epoxy asphalt binder (BEAB), aimed at enhancing the durability and strength of porous asphalt concrete. The experimental results demonstrated that maleic anhydride (MA) was the superior curing agent. The optimized BEAB formulation comprised ESBO, MA, and base bitumen at mass ratios of 7%, 5%, and 88%, respectively. Chen et al. [17] designed three multilayer porous asphalt pavement structures with equivalent thicknesses to actual road pavements. Specimens were thermally insulated using high-performance insulation materials and subjected to controlled-temperature ovens to investigate temperature propagation patterns within the structural layers. The experimental results revealed that: The stabilized internal temperature of specimens was 5–10 °C lower than the ambient temperature, exhibiting a decreasing thermal gradient from the surface layer to the base course, and porous asphalt concrete demonstrated slower temperature conduction rates than conventional dense-graded asphalt concrete. Beatrice et al. [18] employed a resin-based transparent binder combined with light-colored aggregates in an open-graded design to mitigate urban heat island effects. Experimental results demonstrated that incorporating 50% construction and demolition waste aggregates into porous asphalt concrete enhanced both abrasion resistance and mechanical performance. Hanif et al. [19] utilized X-ray Computed Tomography (CT) scanning to quantify microstructural characteristics and sound absorption properties under multiple clogging cycles. They further proposed a non-local means filter to minimize white noise interference, thereby improving image segmentation accuracy. Microstructural analysis revealed severe clogging-induced densification in the top layer (approximately one-third of the sample height), leading to significant alterations in physical pore architecture and void properties [20].

In summary, current researchers have conducted a great deal of research on epoxy bitumen and permeable pavements. However, these studies have not considered the impact of different epoxy bitumen mix ratios on modified bitumen, and there is insufficient research on the pavement performance and functional characteristics of permeable mixtures with larger void ratios, especially the anti-clogging and recovery capabilities of large-void permeable pavements. Therefore, this study focuses on developing an epoxy bitumen and a large-void porous asphalt concrete (LV-PAC) and systematically evaluating its mechanical and functional performance through laboratory investigations. Particular attention is given to drainage efficiency, clogging resistance, and permeability recovery, which remain insufficiently addressed in existing studies on epoxy-modified porous asphalt concrete.

## 2. Materials and Methods

### 2.1. Raw Materials

(1) Epoxy resin

The epoxy binder utilized in this study is composed of two components. Component A is a blend of epoxy resin, toughener, and diluent at a mass ratio of 84:7:9, manufactured by Wanhua Chemical Group (Yantai, China) as product WANNATE 6195, with technical specifications as detailed in Table 1. Component B is a polyamide curing agent A-2, manufactured by Wanhua Chemical Group as product WANNATE 890, with characterization parameters provided in Table 2.

When epoxy resin components are mixed at stoichiometric equivalence, incomplete reaction between amine curing agents and epoxy groups occurs due to steric hindrance within the developing three-dimensional cross-linked network, leaving residual unreacted functional groups. Our prior research demonstrates that epoxy group consumption reaches completion only when the amine curing agent exceeds stoichiometric proportions. Consequently, an optimal mass ratio of Component A:Component B = 100:50 was established to ensure full epoxy group conversion. The general chemical structure of polyurethane synthesized from polyisocyanate (Component A) and polyol together with a chain extender (Component B) is represented as (-NH-COO-)_n_.

The epoxy binder fabrication protocol comprises: (1) Conditioning Component A and Component B separately in a 60 °C oven until softened. (2) Weighing predetermined masses according to the 100:50 ratio. (3) Gradually introducing Component B into Component A under continuous stirring with a glass rod until homogenization. The synthesis of polyurethane was validated through tensile testing. Following synthesis, dumbbell-shaped specimens measuring 6 × 115 mm were prepared using a cutter die. The thickness of each specimen was measured with a vernier caliper prior to testing. The specimens were then conditioned in a constant-temperature chamber at 23 ± 2 °C for 2 h. Subsequently, tensile tests were conducted using a universal testing machine(UTM-100, manufactured by Earth Products China, Hong Kong, China). Prior to each test, the measured thickness was input as a testing parameter, and the crosshead speed was set to 500 mm/min.

Technical specifications of the resultant epoxy binder are provided in Table 3.

According to the Specifcations for Design and Construction of Pavement on Highway Steel Deck Bridge (JTG/T 3364-02-2019) [25], the hot-mix epoxy bitumen is required to have a tensile strength at 23 °C of not less than 2.0 MPa and an elongation at break of no less than 100%. The polyurethane synthesized in this study meets these requirements.

(2) Bitumen

This study employed 70# Kunlun bitumen, Technical specifications were tested in accordance with Standard test methods of bitumen and bituminous mixtures for highway engineering (JTG 3410-2025) [26], with results presented in Table 4

(3) High-Viscosity modified bitumen

This study employed a high-viscosity modified bitumen prepared with a rubber–plastic composite modifier independently developed by the research group. The technical specifications of the material are provided in Table 5.

(4) Aggregate

The coarse aggregates comprised basalt crushed stone with nominal sizes of 10–15 mm and 5–10 mm. Fine aggregates consisted of clean, dry, unweather manufactured sand with appropriate gradation. The filler was limestone-derived mineral powder processed into a hydrophobic material. Technical properties were tested according to Standard Test Methods of Aggregate for Highway Engineering (JTG 3432-2024) [27], with results detailed in Table 6 (coarse aggregate), Table 7 (fine aggregate), and Table 8 (mineral powder).

(5) Fiber

Polyester fibers were selected due to their superior hydrolysis resistance and broad applicability—characteristics particularly advantageous for the developed large-void drainage system. A dosage of 1.0% by mixture mass was implemented [28]. Technical specifications of the polyester fibers are provided in Table 9.

(6) Sealant

Referring to practical conditions, clay was selected as the sealing material. The clay was first dried and compacted into a powdered form, then sieved through 0.3 mm and 0.075 mm meshes. The fractions retained on each sieve were mixed at a 1:1 ratio to obtain the sealant.

### 2.2. Preparation Method of Epoxy Asphalt

**Figure 1:**
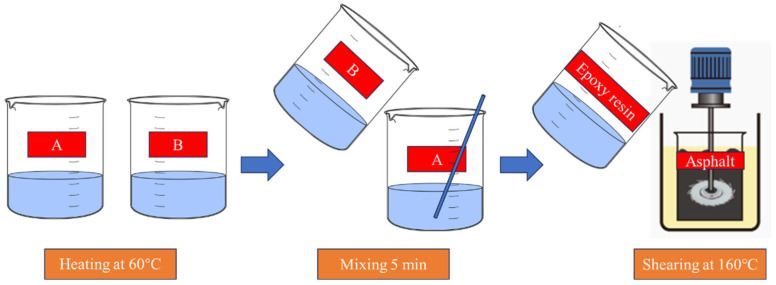
Preparation process of epoxy asphalt.

Epoxy resins, classified as thermosetting polymers, exhibit limited storage stability once compounded with asphalt due to progressive curing reactions. Consequently, epoxy-modified asphalt binders are typically formulated immediately before application. Building on our research group’s prior methodologies [33,34,35], the fabrication sequence for the epoxy asphalt binder in this study follows three critical stages (see Figure 1 for workflow): 1. Thermal Conditioning: Components A (epoxy resin) and B (curing agent) were conditioned in a 60 ± 1 °C forced-air oven until achieving uniform fluidity. 2. Stoichiometric Blending: Component B was gradually transferred into Component A at the predetermined mass ratio (2:1) under continuous mechanical stirring at 200 rpm using a glass rod (manufactured by Longteng Photothermal, Taizhou, China), continuing until optical homogeneity was attained. 3. Asphalt Integration and Homogenization: The blended epoxy system was incorporated into pre-heated 70# base asphalt within a 160 ± 0.5 °C thermostatically controlled mixing chamber. High-shear mixing (1500 rpm) was maintained for 45 ± 5 min to ensure complete molecular-scale dispersion prior to curing initiation.

### 2.3. Test Methods

**Figure 2:**
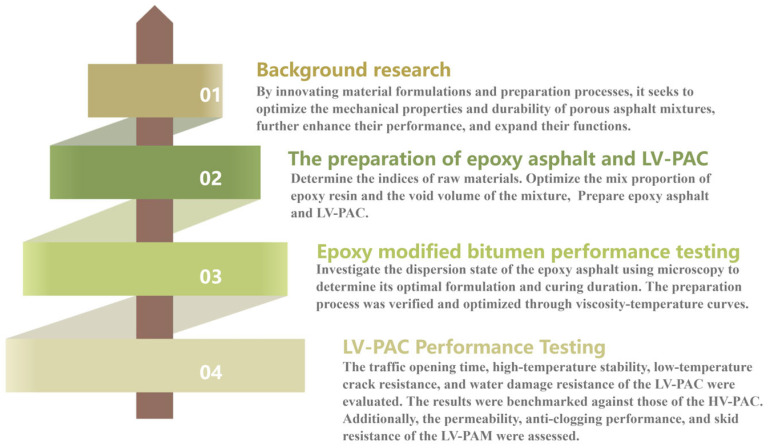
Flowchart of this study.

The test was based on the Chinese standard JTG 3410-2025 [26]. The technical flowchart is shown in Figure 2.

#### 2.3.1. Epoxy Asphalt Tests

(1) Microstructural characterization of epoxy-modified asphalt

Using optical microscopy to determine the optimal formulation ratio, the microstructural characteristics of epoxy asphalt composites were systematically investigated at epoxy resin to base asphalt ratios of 1:3, 1:2, and 1:1. Subsequently, the dispersion evolution of the composite material under varying curing durations was quantitatively analyzed to establish the optimal application timeframe. This comprehensive investigation provides critical insights into the phase distribution characteristics and curing kinetics of the epoxy–asphalt system.

(2) Asphalt viscosity

The Brookfield viscosity was tested using a rotational viscometer (manufactured by Shanghai Changji Geological Instrument Co., Ltd., Shanghai, China). The sample was held at the test temperature for 90 min, after which it was tested. The torque moment was controlled at 10%~98% at temperatures of 150 °C, 160 °C and 170 °C.

#### 2.3.2. LV-PAC Engineering Application Tests

The uniformly mixed epoxy asphalt concrete was immediately transferred into an enamel pan. The pan was then placed in an oven maintained at 160 °C for curing periods of 1 h, 1.5 h, 2 h, 2.5 h, and 3 h, respectively. Upon completion of the curing process, the mixture was promptly placed into molds and compacted into specimens. These specimens were then allowed to cure at room temperature for 16 h before demolding. The volume and mechanical properties of the specimens were measured promptly thereafter.

#### 2.3.3. Porous Asphalt Concrete Road Performance Tests

(1) Wheel tracking test

The objective is to mold a 300 mm × 300 mm × 50 mm rectangular specimen by rolling compaction method. The wheel tracking test is a simulation of the rutting depth of a pavement, whereby loads are driven on the pavement at 60 °C.

(1) $$DS=\frac{\left(t_{2}-t_{1}\right)\times N}{d_{2}-d_{1}}\times C_{1}\times C_{2}$$

where *DS* is the dynamic stability of asphalt concrete (times/min), the deformation at time *t*_1_ is represented by *d*_1_ (mm), and the deformation at time *t*_2_ is represented by *d*_2_. The round-trip speed of the test wheel, *N*, is typically 42 times/min. Finally, *C*_1_ and *C*_2_ are constants.

(2) Three-Point bending test

A universal testing machine (UTM) was employed to conduct this investigation. The objective is to mold a 250 mm × 30 mm × 35 mm beam specimens by cutting rectangular specimens. The experimental protocol required precise temperature control, with the testing environment maintained at −10.0 °C ± 0.1 °C throughout the procedure. Specimens were subjected to three-point bending deformation under displacement-controlled conditions at a constant crosshead speed of 50 mm/min.

(2) $$S_{B}=\frac{R_{B}}{\varepsilon_{B}}$$

where *S_B_* is the flexural modulus of strength at failure of the specimen (MPa), *R_B_* is the flexural and tensile strength of the specimen at breakage (MPa), and *ε_B_* is the maximum bending and tensile strain of the specimen at breakage.

(3) Marshall stability test

The prepared Marshall specimens were placed in a water bath maintained at 60 °C for 30 min. Then an automatic Marshall tester was used to conduct a test at a loading speed of 50 mm/min. The residual Marshall stability ratio was calculated as follows:

(3) $$R_{s}=\frac{M_{S1}}{M_{S}}\times 100$$

where *R_s_* is residual Marshall stability ratio (%), the stability value for 30 min in water is designated as *M_S_* (kN), while the stability value for 48 h in water is designated as *M_S_*_1_ (kN).

(4) Freeze–thaw splitting test

One set of specimens was then placed in a refrigerator at −18 °C for 16 h, followed immediately by a 60 °C water bath for 24 h. The specimens were immersed in water at a temperature of 25 °C for 2 h, and then subjected to the tester with a loading rate of 50 mm/min.

(4) $$TSR=\frac{R_{T2}}{R_{T1}}\times 100$$

where *TSR* is the strength ratio of freeze–thaw splitting test (%), the *R_T_*_2_ is the average indirect tensile strength of the second group of valid specimens after freeze–thaw cycle (MPa), and *R_T_*_1_ is the average value of indirect tensile strength of the first group of effective specimens without freeze–thaw cycle (MPa).

#### 2.3.4. Porous Asphalt Concrete Functional Tests

(1) Water permeability test

The permeability coefficient serves as a critical performance indicator for porous asphalt concrete, quantifying its drainage capacity under hydrodynamic conditions. This investigation employed clay particles (0.075–0.3 mm gradation) as artificial clogging media to simulate in-service pore occlusion mechanisms. Three prototype high-void-content drainage asphalt concretes with distinct void volumes (20.3%, 23.6%, and 25.8%) were subjected to two sequential clogging cycles to evaluate post-occlusion permeability decay. Subsequent rehabilitation using high-pressure water jet cleaning (0.8 MPa, 15° nozzle angle) enabled comparative analysis of permeability recovery. The experimental protocol incorporated the following theoretical formulation for permeability coefficient calculation.

(5) $$C_{w}=\frac{V_{2}-V_{1}}{t_{2}-t_{1}}\times 60$$

where *C_w_* is permeability coefficient of the asphalt concrete specimen (ml/min), *V*_1_ is initial water volume recorded at first measurement (typically 100 mL), *V*_2_ is final water volume recorded at second measurement (typically 500 mL), *t*_1_ is elapsed time at first measurement (s), and *t*_2_ is elapsed time at second measurement (s).

(2) Pavement skid resistance test

The skid resistance performance of porous asphalt concrete was evaluated through dual methodologies: (a) British Pendulum Tester measurements for dynamic friction coefficient quantification, and (b) volumetric sand patch testing for macrotexture depth characterization. This integrated approach provides complementary metrics of surface microtextured frictional properties and macro-scale drainage capacity critical for wet-pavement safety analysis.

(6) $$F=\frac{WgHZ}{PDp}\times 100$$

where *F* is the British Pendulum Number (BPN), *W* is the calibrated pendulum mass (kg), *H* is the centroidal distance from the oscillation axis (mm), *Z* is the vertical displacement below the null position (mm), *g* is the local gravitational acceleration (m·s^−2^), *P* is the normal static pressure (N), *D* is the effective sliding length (mm), and *p* is the pointer tip rotation radius (mm).

(7) $$TD=\frac{4\times 100\times \;V}{{\pi D}^{2}}$$

where *TD* is the macrotexture depth of the asphalt concrete surface (mm); *V* is the calibrated sand volume (cm^3^), standardized as 25 cm^3^ ± 0.5 cm^3^; and *D* is the mean spread diameter (mm).

## 3. Results and Discussion

The primary motivation for using epoxy asphalt as the binder in this study is to address the limitations of conventional high-viscosity asphalt in achieving adequate drainage capacity under high-intensity rainfall. Epoxy resin was introduced to modify the asphalt binder for three main purposes: (1) to significantly increase the softening temperature, thereby enhancing high-temperature stability and resistance to permanent deformation; (2) to improve adhesive properties, ensuring better bonding between aggregates and providing sufficient mechanical strength even at high air void contents; and (3) to obtain a binder grade with superior durability and moisture resistance, which is essential for large-void porous asphalt mixtures subjected to severe environmental and traffic conditions. These modifications enable the designed mixture to achieve a target air void content of approximately 25% while maintaining satisfactory overall performance.

### 3.1. Properties of Epoxy-Modified Bitumen

#### 3.1.1. Microscopic Characteristics

**Figure 3:**
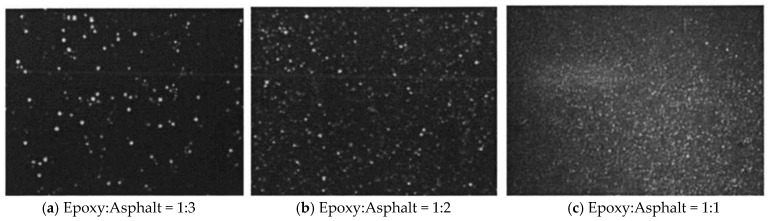
Epoxy resin dispersibility under different proportions.

Optical microscopy observation enables characterization of the microstructural morphology in epoxy-modified asphalt binders, facilitating analysis of phase evolution and real-time monitoring of curing kinetics. This provides critical feedback for formulation optimization and process parameter refinement [36,37]. Figure 3 illustrates the dispersion homogeneity of epoxy resin domains at varying compositional ratios.

Figure 3 demonstrates homogeneous dispersion of epoxy resin across all three compositional ratios within the base asphalt matrix. The spatial density of epoxy domains increases inversely with base asphalt content. At an epoxy-to-asphalt ratio of 1:3, epoxy phase scarcity results in isolated domains (Figure 3a), yielding insufficient crosslinking density for effective asphalt modification. When the ratio reaches 1:2, increased epoxy domain density remains inadequate (Figure 3b), preventing formation of a continuous crosslinked network. At the critical 1:1 ratio, epoxy phases dominate the microstructure (Figure 3c), enabling continuous curing reactions that establish a percolating crosslinked network. This definitive dispersion analysis establishes 1:1 epoxy-to-asphalt as the optimal formulation [38].

**Figure 4:**
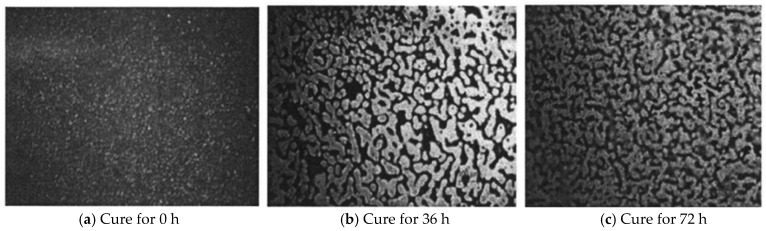
Curing state of epoxy-modified bitumen with different curing times.

To investigate temporal evolution of the 1:1 formulation at 60 °C, fluorescence microscopy tracked curing progression over time (Figure 4). The sequence reveals progressively evolving crosslinked morphology, confirming network development kinetics.

Figure 4 delineates the microstructural evolution of the epoxy–asphalt binder during isothermal curing at 60 °C. At 0 h (Figure 4a); uncured epoxy resin manifests as discrete spherical particles uniformly dispersed within the asphalt matrix, confirming quiescent reaction kinetics. Upon reaching 36 h (Figure 4b), epoxy domains undergo coalescence-driven morphological transition from particulate to dendritic network structures while remaining predominantly asphalt-encapsulated, indicating subcritical crosslinking conversion. Ultimately at 72 h (Figure 4c), phase inversion yields a percolating three-dimensional network through spinodal decomposition, where continuous epoxy phases replace discrete dispersions [39].

#### 3.1.2. Viscosity Properties

During epoxy-modified bitumen fabrication, temperature critically governs workability and aging kinetics [40,41]. Excessively low temperatures elevate viscosity, impeding homogeneous mixing, while elevated temperatures accelerate oxidative aging. Optimal temperature selection is therefore essential. For the high-temperature-resistant epoxy-modified bitumen developed in this study (requiring mixing temperatures >150 °C), viscosity evolution was quantified using a Brookfield viscometer at 150 °C, 160 °C, and 170 °C.

**Figure 5:**
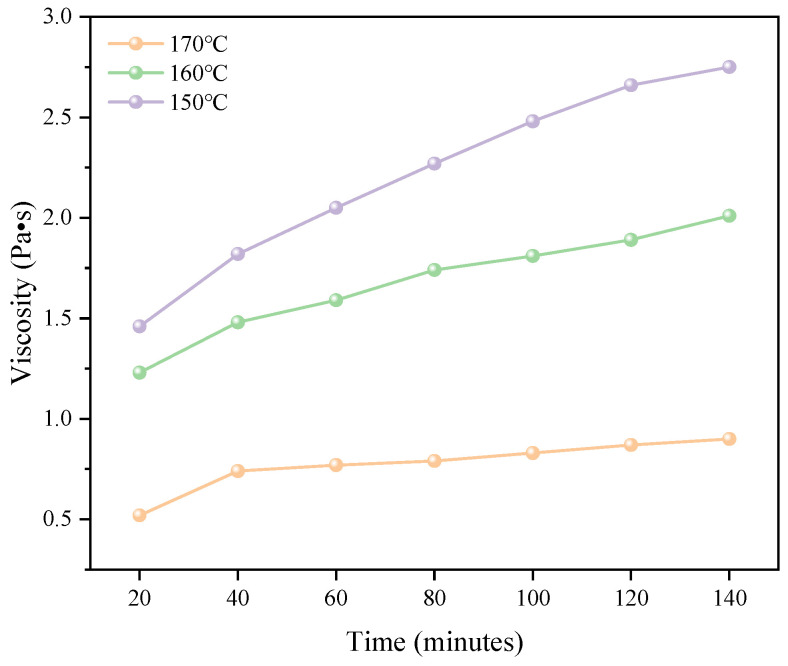
Epoxy-modified bitumen viscosity–temperature curve.

As evidenced in Figure 5, viscosity progressively increased over time at all temperatures, exhibiting inverse temperature dependence: Initial viscosity escalation was rapid, reaching 1.5 Pa·s at 150 °C and 1.25 Pa·s at 160 °C within approximately 20 min; beyond 40 min, viscosity growth curves flattened though continued rising; and terminal viscosities at 2 h measured 2.66 Pa·s (150 °C), 1.89 Pa·s (160 °C), and 0.87 Pa·s (170 °C). The 170 °C condition was eliminated due to accelerated aging kinetics, while 150 °C yielded prohibitively high viscosity that would impede adequate mixing. Consequently, 160 °C was selected as the optimal mixing temperature, balancing manageable viscosity (<2.0 Pa·s).

### 3.2. Engineering Applicability of LV-PAC

#### 3.2.1. Mix Proportion Design of Large-Void Porous Mixture

**Figure 6:**
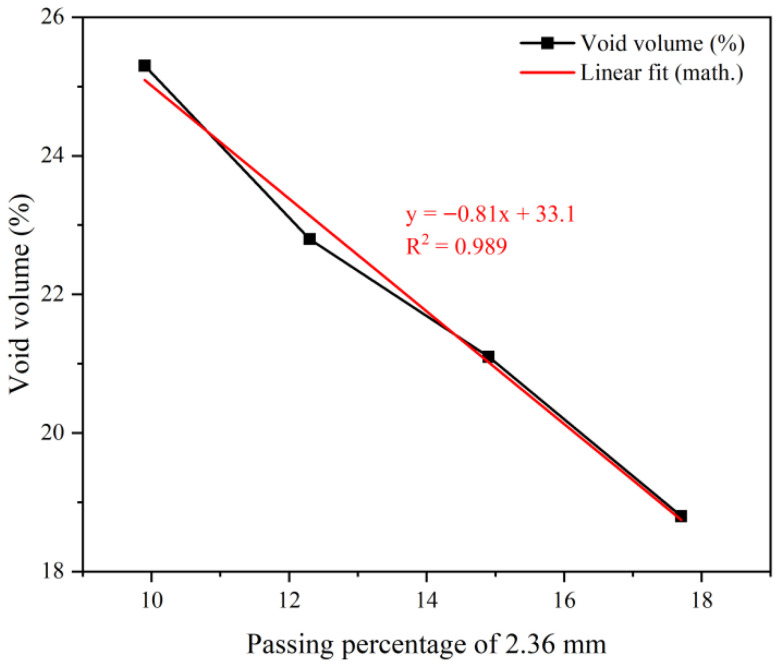
Relationship between critical sieve passage percentage and void volume.

**Figure 7:**
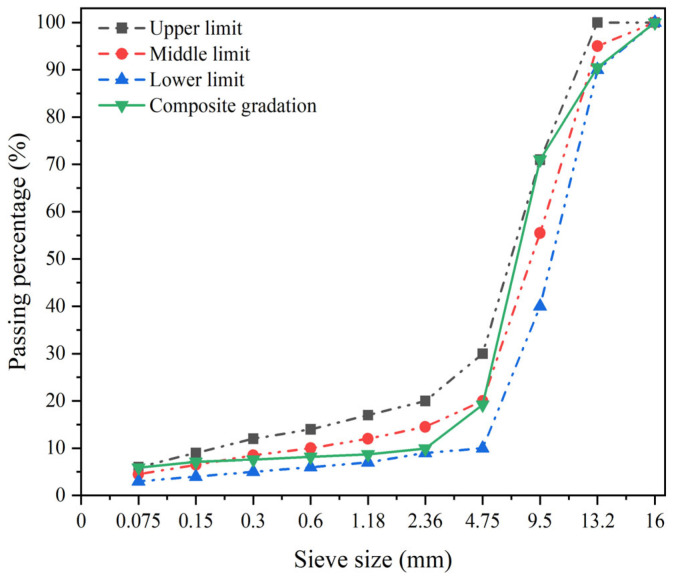
Gradation curve of the PAC-13–type mixture.

Research indicates a significant correlation between percent passing of key sieve sizes and air void content [42]. For large-void epoxy-modified porous asphalt concrete, the influence of key sieve passing rates on void formation requires systematic investigation [43]. Accordingly, this study designed four gradations with 3% differentials in key sieve passing rates following the Technical Specifications for Design and Construction of Porous Asphalt Pavement (JTG/T 3350-03-2020) [44]. The asphalt content was determined via theoretical calculation method assuming a uniform asphalt film thickness of 14 μm, as shown in Figure 6. Air void content decreased progressively with the increasing 2.36 mm sieve passing rate. At 9.9% passing for the 2.36 mm sieve, the measured void content was 25.8%. This establishes that void contents exceeding 25% require <9.9% passing at 2.36 mm. Given the target void content of 25% for our large-void epoxy-modified porous asphalt concrete, the 2.36 mm sieve passing rate must not exceed 9.9%. The finalized PAC-13 gradation curve meeting this criterion is presented in Figure 7.

The optimal asphalt content was determined through draindown and Cantabro loss tests. To enhance asphalt film thickness and consequently improve pavement strength in the LV-PAC, a higher asphalt content of 4.8% was selected as the optimal ratio. The gradation profile shown in Figure 7 was adopted. Key fabrication steps comprised the following: 1. Fiber-Aggregate Preblending: Polyester fibers (1.0% by mix mass) were introduced into preheated aggregates (180 °C) and dry-mixed for 90 s to ensure uniform distribution. 2. Epoxy Binder Formulation: The epoxy resin system (Component A:B = 100:50) was blended with heated asphalt binder (160 °C) under high-shear mixing (1500 rpm, 120 s) to achieve molecular-scale integration. 3. Coating Phase: The epoxy-modified asphalt binder was combined with fiber-treated aggregates in a thermostatic mixer (170 °C), mixing for 90 s until ≥95% coating efficiency was attained. 4. Filler Incorporation: Mineral filler was incrementally added during the final 90 s of mixing to prevent agglomeration while ensuring complete homogenization.

#### 3.2.2. Initial Setting Time

**Figure 8:**
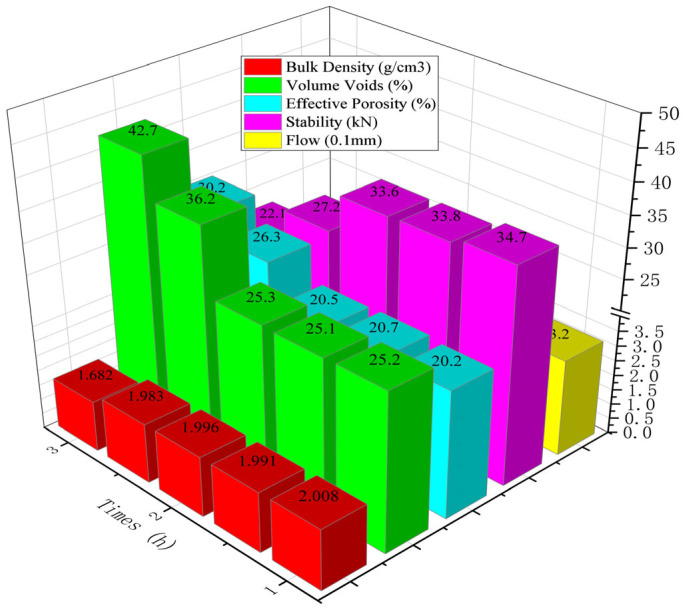
Relationship between curing time and volumetric and mechanical indicators of LV-PAC.

The curing process of epoxy resin exhibits time-dependent behavior analogous to that of mixtures. Therefore, investigating the impact of curing time during transportation on mixture compaction is of critical importance [15]. In this section, a high-temperature drying oven with forced air circulation was employed to simulate the thermal insulation conditions during construction and transportation. The experimental results are presented in Figure 8.

As shown in Figure 8, under identical compaction conditions, the void content of the specimens gradually increased with extended curing time due to the rising viscosity of the system. A particularly sharp increase was observed between 2 h and 2.5 h, where the void content rose from 25.3% to 36.2%, representing a growth rate of 42.3%. This can be attributed to the rapid viscosity development of the epoxy-modified bitumen during this period. The rheological behavior of the epoxy-modified bitumen is characterized by a relatively slow viscosity increase during the initial mixing phase, which has negligible impact on the compaction of the asphalt concrete. However, after 2 h of curing, the viscosity accelerates markedly, resulting in a sharp rise within a short timeframe. Higher viscosity directly leads to greater void content under the same compaction energy. The increased void content indicates reduced compaction efficiency, which is further supported by the changes in Marshall stability: the stability remained largely stable before 2 h but began to decrease rapidly thereafter.

It can therefore be concluded that the compaction of epoxy asphalt concrete should be completed within 2 h after mixing. Considering practical construction processes such as mixing, transportation, paving, and rolling, it is recommended that the entire process from mixing to final compaction of porous epoxy asphalt concrete should not exceed 2 h.

#### 3.2.3. Rapid Opening to Traffic

**Figure 9:**
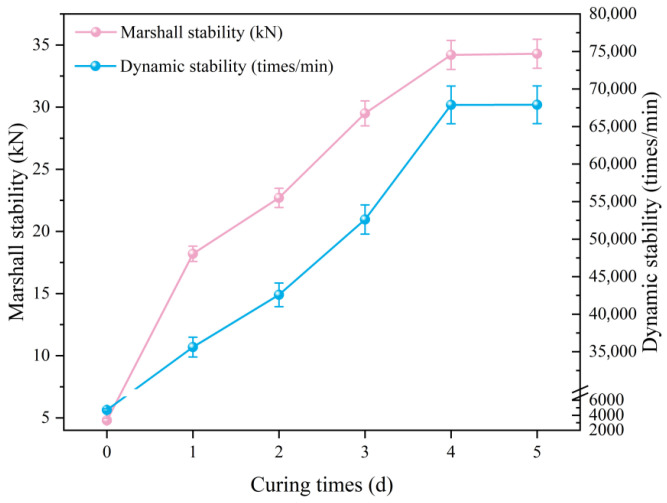
Relationship between specimen curing time and Marshall stability and rutting dynamic stability.

The strength of LV-PAC increases progressively through binder curing post-compaction, necessitating controlled curing under paving conditions. While Jia et al. [45] employed 130 °C curing for polyurethane-modified mixtures monitored via Marshall stability development, such high-temperature curing is impractical for field implementation. Moreover, reliance on a single mechanical indicator may introduce evaluation inaccuracies. To address these limitations, this study implemented a 60 °C curing environment simulating realistic field conditions. This protocol enables quantification of: curing degree development under service-relevant temperatures; multi-parameter strength evolution (Marshall stability, dynamic stability). Experimental results delineating curing progression are presented in Figure 9.

Based on rigorous analysis of the curing kinetics presented in Figure 9, the Marshall stability and dynamic stability of LV-PAC exhibit rapid enhancement during early curing stages, followed by progressively diminishing growth rates after Day 3. The mechanical properties approach a plateau by Day 4, achieving peak values of 35.3 kN (Marshall stability) and 67,512 cycles/mm (dynamic stability). Subsequent testing through Day 5 confirmed negligible further strength development. Critical performance thresholds emerge at 50% curing degree, where the mixture develops sufficient fatigue resistance for traffic loading. These findings establish 3 days post-paving as the optimal traffic-opening timeframe, balancing infrastructure utilization efficiency with material performance requirements.

### 3.3. Pavement Performance of Porous Asphalt Concrete

#### 3.3.1. High-Temperature Stability

**Figure 10:**
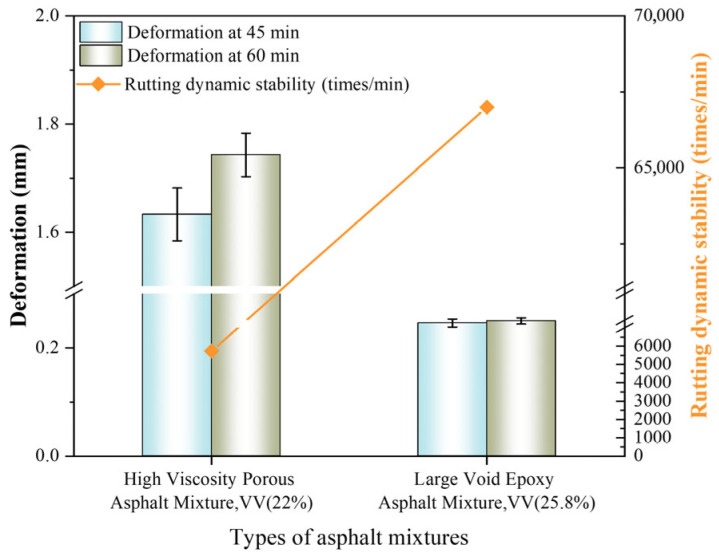
Results of wheel tracking test.

The wheel tracking test replicates asphalt pavement behavior under summer high-temperature and heavy-load conditions through repeated loading wheel passes. It quantifies rutting resistance by measuring permanent deformation per unit load cycle, where higher dynamic stability (DS) values indicate superior high-temperature performance [46]. The deformation and dynamic stability of the two types of porous asphalt concrete are shown in Figure 10.

As shown in Figure 10, both porous asphalt concretes exceeded the minimum DS requirement of ≥5000 cycles/mm. However, significant performance divergence was observed. HV-PAC: DS = 5727 cycles/mm, while LV-PAC: DS = 67,512 cycles/mm. This represents an 11.8-fold enhancement over the HV-PAC and 13.5-fold improvement versus the specification threshold. The exceptional performance stems from the thermosetting nature of epoxy-modified bitumen, where irreversible crosslinking between epoxy resin and curing agent forms a high-strength network. This microstructure maintains dimensional stability at elevated temperatures, demonstrating epoxy-modified bitumen’s profound efficacy in enhancing rutting resistance for LV-PAC [46].

#### 3.3.2. Low-Temperature Crack Resistance

**Figure 11:**
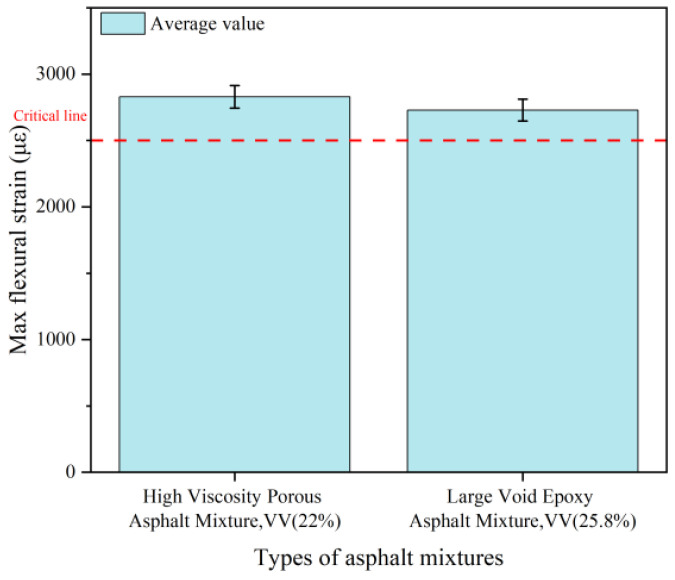
Results of three-point blending test.

The beam bending test at low temperatures characterizes asphalt concrete performance through maximum flexural tensile strain, flexural strength, and stiffness modulus-critical parameters for evaluating low-temperature ductility and embrittlement susceptibility in porous asphalt [47]. Figure 11 presents comparative results for both mixtures.

The LV-PAC exhibited 2729 με maximum flexural strain, marginally lower than the 2830 με of high-viscosity modified mixture, yet compliant with specification requirements. This divergence stems from fundamental material transformations. Firstly, epoxy-modified bitumen’s irreversible 3D crosslinked network converts asphalt from thermoplastic to thermosetting, elevating strength and stiffness while constraining molecular chain mobility. This network architecture inherently limits plastic deformation capacity [48]. High-viscosity modifiers (rubber-plastic composites) enhance ductility via physical crosslinks and elastic networks, expanding the viscoelastic strain range by 35–40% and reducing phase angle peaks by 15–20° under dynamic loading. Secondly, Epoxy systems demonstrate brittle fracture propensity, where stress concentration initiates rapid surface microcrack propagation with limited crosslink-induced crack resistance. HV-PAC exhibits ductile energy dissipation: flexible components absorb fracture energy through elastic deformation, extending fatigue life 3.2-fold versus high-viscosity asphalt while sustaining higher pre-failure strains [49]. Critically, the LV-PAC 25.8% air void content (vs. 22% in HV-PAC) further influences stress distribution and crack initiation pathways.

#### 3.3.3. Water Damage Resistance

(1) Residual Marshall stability

**Figure 12:**
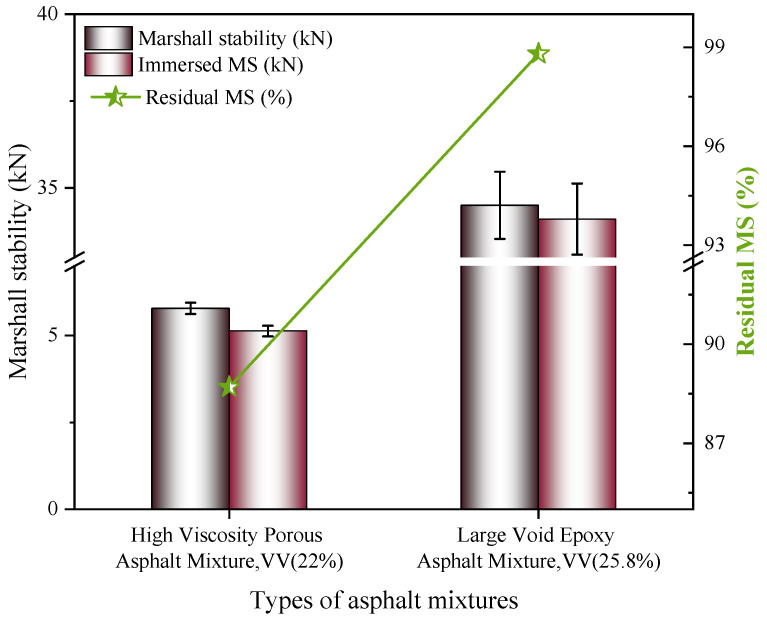
Results of Marshall test.

The test results of residual stability of the two types of asphalt concrete are shown in Figure 12.

Due to the crosslinked network formed in epoxy-modified asphalt, the material exhibits exceptional resistance to shear deformation [50]. Marshall test results indicate that the large-void porous asphalt concrete (LV-PAC) requires a failure load 497% higher than that of the control mixture, attributed to its enhanced aggregate skeleton stability. In terms of moisture damage resistance, the ultra-low surface free energy of epoxy resin effectively suppresses water penetration into the asphalt–aggregate interface. Furthermore, covalent bonding between epoxy and surface hydroxyl groups on aggregates generates interfacial strength substantially greater than that achieved through physical adsorption in polymer-modified binders. As a result, the retained Marshall stability of epoxy-modified asphalt reaches 98.8%, which is 10.1 percentage points higher than the 88.7% observed for HV-PAC.

(2) Indirect tensile strength ratio

**Figure 13:**
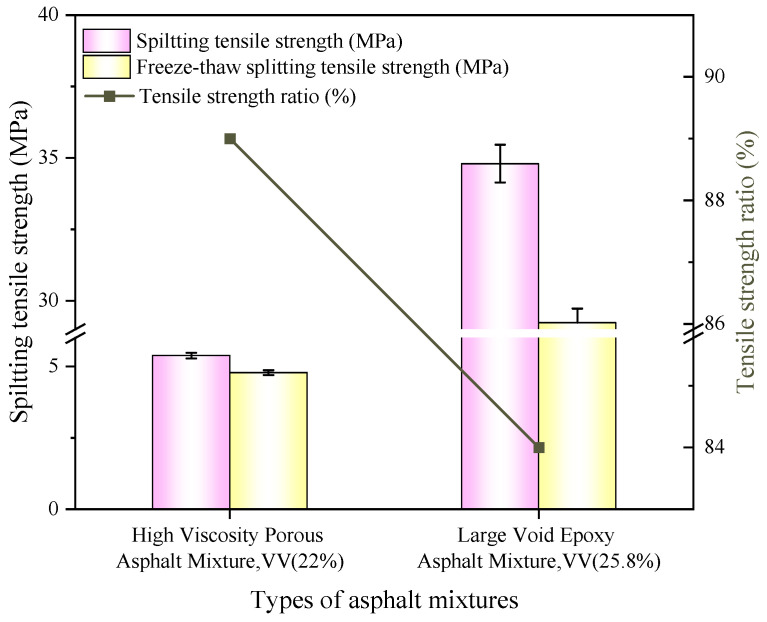
Results of freeze–thaw splitting test.

The test results of freeze–thaw splitting of the two types of asphalt concrete are shown in Figure 13.

The freeze–thaw splitting test, simulating moisture cycling under service conditions, provides a more rigorous assessment of moisture damage resistance than immersion Marshall testing [51]. Figure 13 demonstrates that the LV-PAC achieved a splitting tensile strength of 34.8 MPa—547% higher than HV-PAC (5.38 MPa)—attributable to epoxy’s covalent bonding and enhanced modulus. However, its tensile strength ratio (TSR) of 84% was marginally lower than the HV-PAC’s 89%. This divergence stems from two synergistic mechanisms. 1. Rigidity-induced microcracking: Water saturation in 25.8% voids generates expansive pressure during freezing. The high rigidity of the epoxy network limits plastic deformation, preventing stress dissipation and triggering rapid microcrack propagation. 2. Enhanced water retention: Higher void volume stores significantly more freeze-expandable water, amplifying hydraulic damage [52]. These findings align with beam bending test results confirming the material’s constrained strain capacity at low temperatures.

### 3.4. Functional Characteristics of Porous Asphalt Concrete

The permeability coefficient and skid resistance coefficient constitute the dual functional pillars for evaluating porous asphalt concrete. The permeability coefficient governs drainage efficiency, preventing water damage while supporting sponge city infrastructure. The skid resistance coefficient ensures driving safety, particularly under adverse weather conditions. These functional superiorities distinguish porous asphalt from conventional mixtures and underpin its global adoption in high-grade highways [53,54,55].

#### 3.4.1. Water Permeability

**Figure 14:**
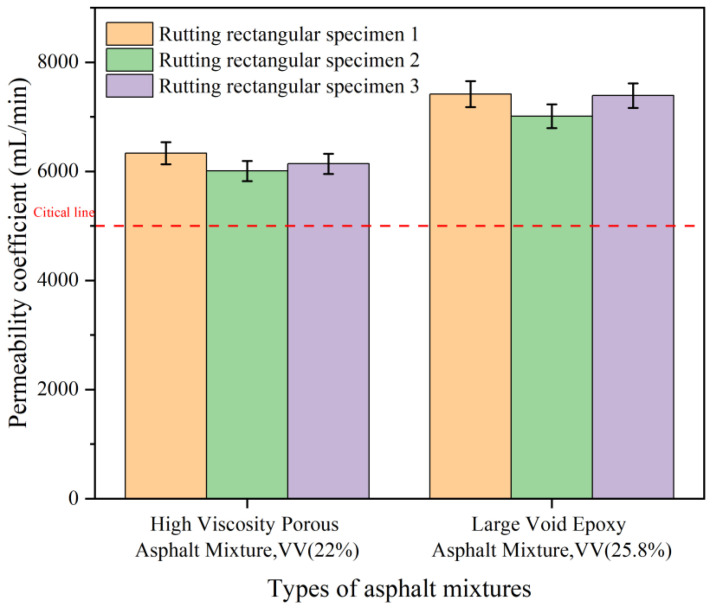
Results of water permeability test.

Three sets of wheel tracking specimens were tested, with three replicates per set. Average values are reported as final results (Figure 14).

As shown in Figure 14, Both mixtures exceeded the permeability specification threshold (>5000 mL/min). The LV-PAC achieved 7272 mL/min-demonstrating 45.4% superiority over specification requirements and 18.1% enhancement versus HV-PAC, confirming exceptional drainage functionality. Implementation of this material enables rapid rainwater permeation through its macrovoid structure, reducing surface runoff by >80%. This significantly mitigates hydroplaning risks, potentially decreasing wet-weather accidents by 22–37%.

#### 3.4.2. Anti-Clogging Property

**Figure 15:**
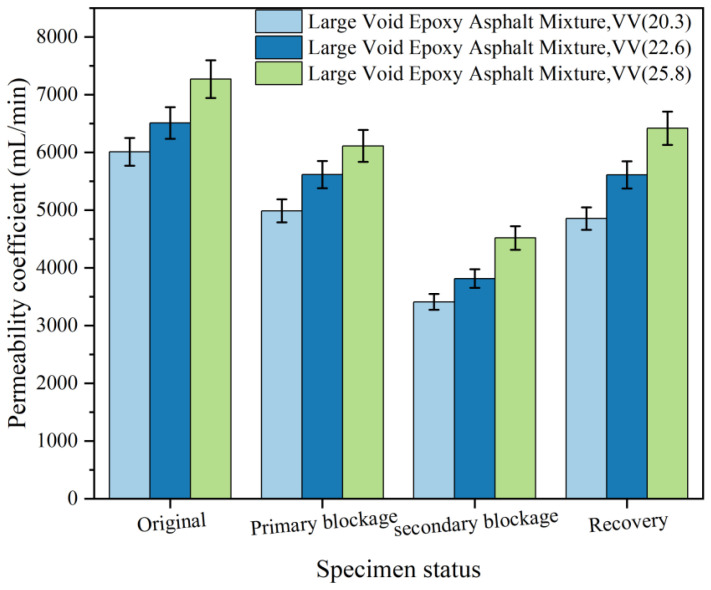
Results of water permeability after clogging recovery test.

HV-PAC exhibits limited drainage efficiency recovery (<65%) after high-pressure flushing—a critical limitation hindering widespread implementation [56,57]. This study subjected LV-PAC specimens with varying void contents (20.3%, 22.6%, 25.8%) to two sequential clogging cycles, measuring permeability after each event (Figure 15). Post-clogging high-pressure water jetting (20 MPa, 30° nozzle) was then applied.

Based on the experimental data presented in Figure 15, the LV-PAC exhibits a significant permeability reduction after two identical clogging cycles, with the second clogging causing a more pronounced decline than the first. This demonstrates that timely cleaning intervention is critical upon initial clogging detection, as delayed maintenance accelerates pore obstruction rates. The permeability reduction shows clear void-content dependency: specimens with 25.8% voids experienced 35% permeability loss, outperforming 22.6% void specimens (40% loss) and 20.3% void specimens (46% loss). The gradual degradation curve at 25.8% void content confirms superior clogging resistance in large-void designs. Post-cleaning recovery efficiency further validates this advantage: 25.8% void mixtures restored 92% of initial permeability—significantly higher than 87% recovery at 22.6% voids and 80% at 20.3% voids. This 12-percentage-point improvement demonstrates optimal hydraulic restoration in high-void systems, attributable to enhanced pore connectivity (>88% vs. 72% in 20.3% voids) and more effective sediment displacement during high-pressure washing. Critically, the accelerated degradation during the second clogging cycle (18–24% greater reduction than initial clogging) underscores the necessity for prompt maintenance protocols. These findings establish 25.8% void content as the optimal threshold for balancing drainage performance and service life in clogging-prone environments.

#### 3.4.3. Anti-Skid Performance

**Figure 16:**
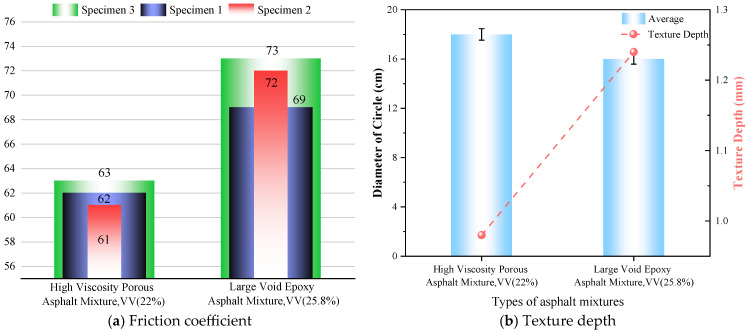
Results of the anti-skid ability of the two asphalt concretes.

The skid resistance coefficient of porous asphalt pavement can be evaluated by the friction coefficient measured with the pendulum tester and the texture depth measured with the sand patch method [58]. The pendulum tester results are shown in Figure 16a, and the texture depth results measured by the sand patch method are shown in Figure 16b.

As shown in Figure 16a, the British Pendulum Number (BPN) of the LV-PAC reaches 71.3. This represents an improvement of over 10% compared to the HV-PAC. This value significantly exceeds the requirement of >45 BPN for expressway pavements specified in Inspection and Evaluation Quality Standards for Highway Engineering Section 1: Civil Engineering [59] (JTG F80/1-2017). These results demonstrate that the LV-PAC, while satisfying the requirement for high-performance drainage, exhibits excellent surface texture. This enables a substantial enhancement in skid resistance compared to HV-PAC. Figure 16b indicates that the texture depths of both the LV-PAC and the HV-PAC meet the specification requirements. Notably, the LV-PAC exhibits superior skid resistance performance to the HV-PAC, showing an improvement of 26.5%. This confirms that the LV-PAC possesses excellent texture depth, contributing significantly to enhanced pavement skid resistance. Pavements with high skid resistance can reduce vehicle braking distances, thereby improving road safety. This improvement can be attributed to the higher air void content inherent in the LV-PAC. Within the range that does not compromise pavement performance or normal vehicle operation, a higher air void content leads to greater texture depth and, consequently, enhanced driving safety.

This improvement can be attributed to the higher air void content inherent in the LV-PAC. Within the range that does not compromise pavement performance or normal vehicle operation, a higher air void content leads to greater texture depth and, consequently, enhanced driving safety.

## 4. Conclusions

This study developed an epoxy-modified bitumen binder and a novel LV-PAC. The mechanical properties and functional characteristics of these materials were compared with those of the most widely used HV-PAC. The conclusions are as follows:

(1) The epoxy-modified bitumen developed in this study is recommended for use at 160 °C. When the epoxy resin to base asphalt ratio is 1:1, a crosslinked network structure forms within the epoxy-modified bitumen, resulting in optimal blending. Upon reaching a curing time of 72 h at 60 °C, the epoxy resin transitions from a dispersed phase to a continuous phase, endowing the epoxy-modified bitumen with high strength. The epoxy-modified bitumen should be used within 72 h of production; beyond this period, the increased viscosity makes mixture compaction difficult. It is recommended that the entire process from mixing to final compaction of porous epoxy asphalt concrete should not exceed 2 h, and the traffic opening time for the LV-PAC is 3 days after paving.

(2) The epoxy-modified bitumen exhibits high strength and adhesion. By controlling key sieve sizes, the air void content of the mixture was increased to 25.8%. The LV-PAC demonstrated outstanding performance in key tests: Wheel Tracking Test: Dynamic stability reached 67,000 passes/mm; Low-Temperature Bending Beam Test: Maximum tensile strain was 2729 με; Marshall Stability Test: Stability value reached 34.5 kN, with a retained Marshall stability after water immersion of 98.8%; Freeze–Thaw Splitting Test: The tensile strength ratio (TSR) was 84%. Notably, the splitting strength reached 34.8 MPa, substantially higher than the 5.38 MPa achieved by the HV-PAC.

(3) Permeability tests yielded the following results: The LV-PAC (25% air voids) had a permeability coefficient of 7272 mL/min. After pore declogging, the permeability coefficient recovered to 92% of its initial value. The permeability coefficient of the LV-PAC was 1113 mL/min higher (an improvement of 18.1%) than that of the HV-PAC (22% air voids), demonstrating superior permeability and exceptional anti-clogging performance. The LV-PAC also exhibited outstanding skid resistance, with a British Pendulum Number (BPN) of 71.3 and a texture depth of 1.23 mm.

(4) Leveraging the high rigidity of its chemical crosslinked network, strong interfacial chemical bonding, and optimized gradation, the LV-PAC exhibited significantly superior compressive strength and moisture stability compared to the HV-PAC. Although the HV-PAC offers advantages in deformation adaptability and construction convenience, its physical modification mechanism and open-graded design result in relatively lower stability indices. In practical engineering, material selection should be based on climatic conditions and functional requirements. While the rigid crosslinked network and strong chemical bonding of the epoxy-modified bitumen impart higher tensile strength to the LV-PAC, its inherent brittleness, susceptibility to moisture retention, and sensitivity to hydrothermal aging lead to more significant performance degradation after freeze–thaw cycles compared to the HV-PAC.

This study developed a novel epoxy-modified bitumen that meets the current industry requirements for high-performance asphalt. This study has certain limitations in evaluating the novel epoxy-modified bitumen for large-void porous asphalt concrete. In future research, we will conduct Hamburg wheel tracking tests under coupled water-temperature conditions, as well as flexural fatigue tests, among others.

## Figures and Tables

**Table 1:** Performance of epoxy resin component A.

Item	Test Value	Test Method
Viscosity (25 °C, mPa·s)	8000–10,000	GB/T 22314 [21]
Epoxy Equivalent (g/eq)	170–190	GB/T 4612 [22]
Density (25 °C, g/cm^3^)	0.995–1.010	GB/T 15223 [23]
Appearance	White transparent viscous liquid	Observed visually

**Table 2:** Performance of epoxy resin component B.

Item	Test Value	Test Method
Viscosity (25 °C, mPa·s)	370–450	GB/T 22314 [21]
Amine value (mg KOH/g)	170–190	GB/T 2895 [24]
Density (25 °C, g/cm^3^)	0.995–1.010	GB/T 15223 [23]
Appearance	White transparent viscous liquid	Observed visually

**Table 3:** Performance of epoxy resin.

Item	Test Value	Test Method
Viscosity (25 °C, mPa·s)	9000–11,000	GB/T 22314 [21]
Epoxy Equivalent (g/eq)	170–190	GB/T 4612 [22]
Density (25 °C, g/cm^3^)	1.0–1.1	GB/T 15223 [23]
Appearance	White transparent viscous liquid	Observed visually
Elongation at break (23 °C, %)	150.8	JTG/T 3364-02-2019 [25]
Tensile strength (23 °C, MPa)	7.1	JTG/T 3364-02-2019 [25]

**Table 4:** Test results of 70# bitumen technical indicators.

Item	Unit	Test Value	Standard Requirement	Test Method
Penetration (25 °C, 100 g, 5 s)	0.1 mm	68	60–80	T 0604
Softening point	°C	48	≥46	T 0606
Ductility (15 °C, 5 cm/min)	cm	>100	≥100	T 0605
RTOFOT				
Change in Residual Mass	%	0.04	≤±0.8	T 0610
Residual Penetration Ratio (25 °C)	%	67.4	≥61	T 0604
Ductility (10 °C)	cm	8.3	≥6	T 0605

**Table 5:** Test results of high-viscosity modified bitumen technical indicators.

Item	Unit	Test Value	Standard Requirement	Test Method
Penetration (25 °C, 100 g, 5 s)	0.1 mm	45	60–80	T 0604
Softening point	°C	96	≥46	T 0606
Ductility (5 °C, 5 cm/min)	cm	41	≥100	T 0605
RTOFOT				
Change in residual mass	%	0.02	≤±0.8	T 0610
Residual Penetration Ratio (25 °C)	%	81	≥61	T 0604
Ductility (5 °C)	cm	29	≥6	T 0605

**Table 6:** Test results of coarse aggregate technical indicators.

Item		Unit	Test Value		Standard Requirement	Test Method
Aggregate particle size			5–10 mm	10–15 mm		
Apparent specific gravity		g/cm^3^	2.953	2.962	≥2.70	T 0304
Bulk specific gravity		g/cm^3^	2.902	2.921	≥2.60	
Water Absorption		%	0.62	0.47	≤1.0	
Soundness		%	3.2	4.9	≤8	T 0314
Content of particles smaller than 0.075 mm		%	0.3	0.2	≤1	T 0310
Flakiness Index	<10 mm	%	3.0	/	≤12	T 0312
	>10 mm		/	1.6	≤10	
Los Angeles Abrasion Loss		%	/	8.95	≤20	T 0317
Aggregate crushing value		%	/	10.0	≤20	T 0316
Adhesion to Bitumen		Level	/	5	≥5	T 0616

**Table 7:** Test results of fine aggregates technical indicators.

Item	Unit	Test Value	Standard Requirement	Test Method
Apparent specific gravity	g/cm^3^	2.742	≥2.60	T 0330
Soundness (>0.3 mm)	%	2.8	≤3	T 0340
Angularity (Flow Time)	s	35	≥30	T 0345

**Table 8:** Test results of mineral powder technical indicators.

Item		Unit	Test Value	Standard Requirement	Test Method
Apparent specific gravity		g/cm^3^	2.733	≥2.60	T 0352
Particle size range	<0.60 mm	%	100.0	100	T 0351
	<0.30 mm		99.9	95–100	
	<0.15 mm		99.6	90–100	
	<0.075 mm		90.4	80–100	
Appearance		/	no agglomeration or caking	no agglomeration or caking	/
Heating stability			no obvious discoloration	actual measurement records	T 0355

**Table 9:** Test results of polyester fiber technical indicators.

Item	Unit	Standard Requirement	Test Method
Heat resistance (210 °C, 2 h)	/	Volume and color show no obvious changes	JT/T 533 [29]
Breaking strength	MPa	≥500	GB/T 3916 [30]
Elongation at break	%	≥15	GB/T 3916 [30]
Length	Mm	9 ± 1	GB/T 14336 [31]
Diameter	μm	15 ± 5	GB/T 10685 [32]

## Data Availability

The raw data supporting the conclusions of this article will be made available by the authors on request.
